# A nomogram for predicting overall survival of patients with sinonasal melanoma: A population‐based study

**DOI:** 10.1002/lio2.951

**Published:** 2022-11-22

**Authors:** Jingyi Yang, Xiaole Song, Yuting Lai, Quan Liu, Xicai Sun, Dehui Wang, Hongmeng Yu

**Affiliations:** ^1^ Department of Otolaryngology, Eye and ENT Hospital Fudan University Shanghai People's Republic of China; ^2^ Mucosal Melanoma Treatment Center, Eye and ENT Hospital Fudan University Shanghai People's Republic of China; ^3^ Research Units of New Technologies of Endoscopic Surgery in Skull Base Tumor Chinese Academy of Medical Sciences Beijing People's Republic of China

**Keywords:** mucosal melanoma, nomogram, overall survival, SEER database, sinonasal melanoma

## Abstract

**Objective:**

Sinonasal melanoma (SMM) is a rare but aggressive malignancy with 5‐year overall survival (OS) rates below 40% in published studies. However, the clinicopathological predictors of the prognosis of SMM remain undefined. We aimed to establish a model to predict the survival outcomes of SMM.

**Methods:**

We searched the Surveillance, Epidemiology, and End Results (SEER) database for patients diagnosed with SMM between 1975 and 2016. Data on patient demographics, treatment modalities, and survival outcomes were retrieved. Risk factors for OS were evaluated by survival and Cox regression analyses. We also developed and validated a nomogram for OS, and compared its performance with that of conventional staging systems.

**Results:**

Overall, 305 SMM patients were included in this population‐based study. Multivariate Cox regression showed that primary site, American Joint Committee on Cancer stage, radiotherapy, and surgery were significant risk factors for survival. A nomogram was established using the regression model. The C‐indices, areas under the receiver operating characteristic curves, calibration plots, and decision curve analysis demonstrated reliable performance of the nomogram.

**Conclusion:**

The nomogram predicting survival outcomes of SMM patients based on clinical information showed good discriminative ability and prognostic accuracy compared with conventional stage classifications. Our nomogram could be used to predict the survival probabilities for SMM patients at different timepoints.

**Level of Evidence:**

2b.

## Graphical abstract

The Surveillance, Epidemiology, and End Results (SEER) database was searched for patients diagnosed with sinonasal melanoma (SMM) between 1975 and 2016. Multivariate Cox regression showed that primary site, AJCC staging, radiotherapy, and surgery were significant risk factors for survival. A nomogram predicting survival outcomes of SMM patients was established and could be used to predict the survival probabilities for SMM patients at different timepoints.

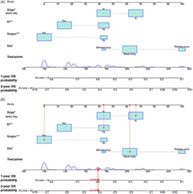

**Abbreviations**
AJCC: American Joint Committee on Cancer
AUC: area under the curve
CI: confidence interval
DCA: decision curve analysis
HR: hazard ratio
IDI: integrated discrimination improvement
NRI: net reclassification improvement
OS: overall survival
ROC: receiver operating characteristic
RT: radiotherapy
SEER: Surveillance, Epidemiology, and End Results
SMM: sinonasal melanoma

## 1 INTRODUCTION

Melanoma is a rare malignancy derived from both cutaneous chromatophores and melanocytes distributed in the mucosal membrane.^1^ Especially, mucosal melanoma which accounts for just 1%–4% of all variants of melanoma originates from the mucosal surface of the head and neck, as well as the digestive and genitourinary tracts.^1^, ^2^ About 50%–55% of mucosal melanomas arise in the head and neck region.^3^ Sinonasal melanoma (SMM) accounts for about 4%–8% of all types of sinonasal malignancies,^4^ with a reported incidence rate of 0.05/100,000 persons/year in the Surveillance, Epidemiology, and End Results (SEER) registry.^3^ Despite its rarity, the incidence of mucosal melanoma increased progressively, especially among diseases originating from the nasal cavity or paranasal sinuses.^5^, ^6^

SMM is a highly aggressive malignancy with 5‐year overall survival (OS) rates of below 40% in published studies.^7^, ^8^ The most common primary locations of SMM are the anterior part of the nasal septum, the inferior turbinate, and the medial wall of the maxillary sinus.^8^ The generalized stage classification proposed by Ballantyne et al. includes three classes/stages based on ascending levels of tumor extension comprising local disease (stage I), regional involvement (stage II), and distant metastasis (stage III).^9^ Ballantyne's stage classification is widely used in clinical practice, but its discriminative ability has been criticized, especially for tumors in advanced stages, due to the scarcity of cervical lymph node metastasis or distant metastasis at initial diagnosis.^7^ The Tumor–Node–Metastasis (TNM) staging system described in the seventh edition of the American Joint Committee on Cancer (AJCC) manual is rarely used for SMM, probably due to the novelty of this staging system.^10^ Additionally, SMM is conventionally treated by surgical resection followed by adjuvant radiotherapy (RT), whereas chemotherapy is usually reserved for advanced cases.^7^, ^11^ A treatment strategy involving a multidisciplinary team approach was recently proposed for SMM.^4^

Nomograms are a visual representation of regression models developed to predict the survival probability at specified timepoints for individual patients with a specific disease. Compared with other predictive statistical methods, nomograms are an intuitive tool for assessing the individual's prognosis. They have been developed and applied to predict the diagnosis or prognosis of numerous types of tumors,^12^, ^13^, ^14^ but none have been developed to predict the survival of SMM patients. Therefore, in this study, we used the SEER registry to develop a nomogram to predict the OS probabilities in SMM patients.

## 2 MATERIALS AND METHODS

### 2.1 Data source

Patient demographics, clinical characteristics, and survival data were collected from the SEER 18 registry of patients diagnosed between 1975 and December 31, 2015 (submission November 2016), using the Case Listing Session of SEER*Stat software (National Cancer Institute, Bethesda, MD; version 8.3.6). The data extraction and analyses were performed in March 2020.

### 2.2 Patient selection and data collection

The International Classification of Diseases for Oncology, Third Edition (ICD‐O‐3) histology/behavior codes for mucosal melanoma comprises melanoma (8720), balloon cell melanoma (8722), mixed epithelioid and spindle cell melanoma (8770), epithelioid cell melanoma (8771), and spindle cell melanoma (8772) according to the World Health Organization (WHO) classification of head and neck tumors. Based on the seventh edition of the AJCC staging system, the ICD‐O‐3 codes for primary sites of SMM include the nasal cavity (C30.0) and paranasal sinuses (C31.0, C31.1, C31.2, C31.3, C31.8, and C31.9). Of note, although the staging criteria for mucosal melanoma remained the same between the seventh and eighth edition of the AJCC staging system, the seventh edition was used for clinical staging since the truncation time of follow‐up (December 31, 2015) in this study was earlier than the publication of eighth edition of AJCC staging in 2017. A 6‐month follow‐up was regarded as the minimum follow‐up duration for these SMM patients. Patients were excluded for the following reasons: (1) cases documented by death certificates or autopsy records only; (2) patients with insufficient clinical data; or (3) patients without definite follow‐up information. The demographic and clinical characteristics extracted from the database included age at diagnosis, race, sex, multiple primary tumor history, pathological subtype, primary tumor site, the seventh AJCC TNM and clinical staging classification, surgical treatment of the primary disease, RT, and chemotherapy. Tumor invasion was defined as stage I (localized disease), stage II (regional cervical lymph node involvement), and stage III (distant metastasis) according to Ballantyne's classification.^9^ OS was defined as the time from diagnosis of SMM to death or the end of follow‐up. Detailed pathologic data, including margin, surgical approach (open or endoscopic), surgical management of the primary site or neck dissection, information about anatomical structure involvement, biological and immunomodulatory oncological interventions, and posttreatment recurrence were inaccessible when extracting data from the SEER registry. A statement of institutional review board approval was waived because of the anonymity of the data utilized and because no direct study on human subjects was included.

### 2.3 Statistical analysis

The entire cohort was randomly divided into a training set and a validation set with a ratio of 2:1. We used *χ* ^2^ tests or Fisher's exact tests to compare categorical variables between the training and validation sets. Survival curves were plotted using the Kaplan–Meier method and compared using the log‐rank test. Univariate Cox regression was performed to investigate the possible predictors of OS, and hazard ratios (HRs) with 95% confidence intervals (CIs) were calculated. Variables with *p*‐values of <.05 in log‐rank tests were identified as possible clinical factors that might influence survival outcomes. The candidate variables were then included in multivariable Cox regression analysis followed by backward stepwise regression to create a model with the smallest Akaike information criterion (AIC). The Cox regression model incorporating these independent significant predictors was interpreted by a nomogram generated using the R package regplot.

Internal cross‐validation was performed to evaluate model performance. The model's discriminative ability was assessed by the C‐index and the area under the curve (AUC) of the receiver operating characteristic (ROC) curves. The C‐indices of different models were compared by the bootstrap method with 1000 resamples. The C‐indices for the nomogram and conventional staging classifications were compared using the R package CompareC. Calibration plots were generated to evaluate the prediction accuracy by bootstrapping with 1000 resamples. Decision curve analysis (DCA) was performed to compare the clinical net benefit of the nomogram and conventional staging systems using the R package stdca. The net reclassification improvement (NRI) and integrated discrimination improvement (IDI) were calculated to show the improvement in the predictive accuracy of the nomogram. All statistical analyses were performed using R software (version 3.5.3; http://www.Rproject.org) with the packages survival, survminer, rms, glmnet, regplot, CompareC, survivalROC, survIDINRI, and stdca. In all tests, two‐sided *p*‐values of <.05 were considered statistically significant.

## 3 RESULTS

### 3.1 Baseline characteristics and overall prognosis

A total of 305 patients pathologically diagnosed with SMM were identified from the SEER registry after applying the inclusion and exclusion criteria. The cohort was divided into the training and validation sets, comprising 204 and 101 patients, respectively. There were no significant differences in baseline characteristics between the training and validation sets (Table 1). Both sets included equal proportions of males and females. The majority of patients in both cohorts were over 70 years old (57.8% in training cohort; 67.3% in validation cohort) and white (87.3% in training cohort; 82.2% in validation cohort). The patients were commonly found with AJCC T3 stage (54.9% in training cohort; 63.4% in validation cohort) and Ballantyne's stage I disease (81.9% in training cohort; 82.2% in validation cohort). The proportions of patients with AJCC stages III or IV were roughly equal. In the training set, most patients received RT (63.7%, 130/204) and surgery (80.9%, 165/204), and a minority of patients received chemotherapy (8.3%, 17/204). The detailed clinical characteristics of the SMM patients are presented in Table 1.

**TABLE 1:** Patient characteristics of the training and the validation cohort

Variables	Category	Training cohort (*n* = 204)		Validation cohort (*n* = 101)		*p* value
		*N*	%	*N*	%	
Age	<50	10	4.9	4	4.0	.285
	50–70	76	37.3	29	28.7	
	>70	118	57.8	68	67.3	
Sex	Male	101	49.5	48	47.5	.838
	Female	103	50.5	53	52.5	
Race	Asian/Pacific Islander	16	7.8	13	12.9	.369
	Black	10	4.9	5	5.0	
	White	178	87.3	83	82.2	
Multiple tumor history	No	133	65.2	65	64.4	.986
	Yes	71	34.8	36	35.6	
Pathology	Melanoma (ICD‐O‐3 code 7020)	196	96.1	93	92.1	.230
	Other pathology subtypes^a^	8	3.9	8	7.9	
Site	Ethmoid sinus	11	5.4	3	3.0	.727
	Maxillary sinus	35	17.2	18	17.8	
	Nasal cavity	158	77.5	80	79.2	
T stage	T3	112	54.9	64	63.4	.199
	T4	92	45.1	37	36.6	
N classification	N0	185	90.7	94	93.1	.816
	N1	15	7.4	6	5.9	
	Nx	4	2.0	1	1.0	
M classification	M0	180	88.2	87	86.1	.736
	M1	24	11.8	14	13.9	
AJCC Clinical stage (seventh)	III	95	46.6	57	56.4	.134
	IV	109	53.4	44	43.6	
Ballantyne stage	I	167	81.9	83	82.2	.419
	II	15	7.4	4	3.9	
	III	22	10.8	14	13.9	
RT	No	74	36.3	42	41.6	.439
	Yes	130	63.7	59	58.4	
Surgery	No	39	19.1	15	14.9	.448
	Yes	165	80.9	86	85.1	
Chemotherapy	No	187	91.7	89	88.1	.431
	Yes	17	8.3	12	11.9	

*Note*: Two‐sided *p* < .05 was considered statistically significant.

Abbreviations: AJCC, American Joint Committee on Cancer; OS, overall survival; RT, radiotherapy; SMM, sinonasal melanoma.

^a^ ICD‐O‐3 codes: 8722, 8770, 8771, 8772.

**FIGURE 1:**
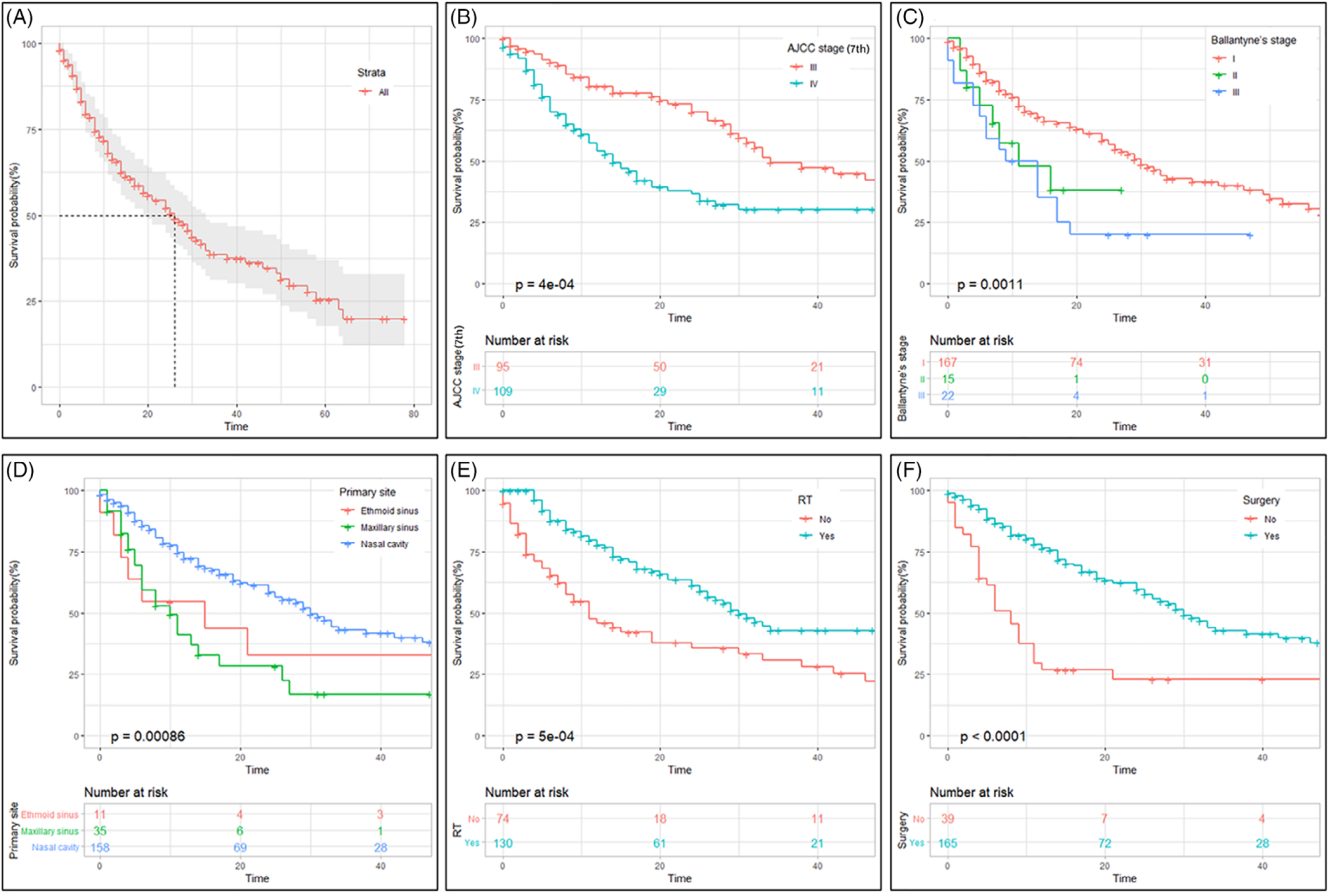
Kaplan–Meier plots of OS for the entire cohort of SMM patients in the training cohort (A) and in patients stratified by seventh AJCC TNM stage (B), Ballantyne's stage (C), primary site (D), surgery (E), and RT (F). AJCC, American Joint Committee on Cancer; OS, overall survival; SMM, sinonasal melanoma; TNM, tumor–node–metastasis

Patients were actively followed up until the date of death or the end of the follow‐up, with a median follow‐up of 15 months (mean: 21.7 ± 19.7 months, range: 1–83 months) in the entire cohort. Overall, 168 patients (55.1%, 168/305) had died by the end of the follow‐up. In the training cohort, the Kaplan–Meier estimated 1‐, 2‐, and 5‐year OS rates were 66.2%, 52.0%, and 25.5%, respectively (Figure 1A). The results of the survival analysis in the training cohort indicated that lower clinical stage (stage III) (Figure 1B) or Ballantyne's stage I (Figure 1C), disease originating in nasal cavity (Figure 1D), lower T stage (T3) (Figure S1A), no cervical lymph node involvement (Figure S1B), no distant metastasis (Figure S1C), surgical treatment (Figure 1E), and RT (Figure 1F) were associated with significantly higher OS rates. More information regarding 1‐ and 2‐year survival rates stratified by these variables were presented in Table S1. No significant survival benefit was observed in patients who received chemotherapy (Figure S1D).

### 3.2 Development of the prognostic nomogram for OS in SMM patients

**FIGURE 2:**
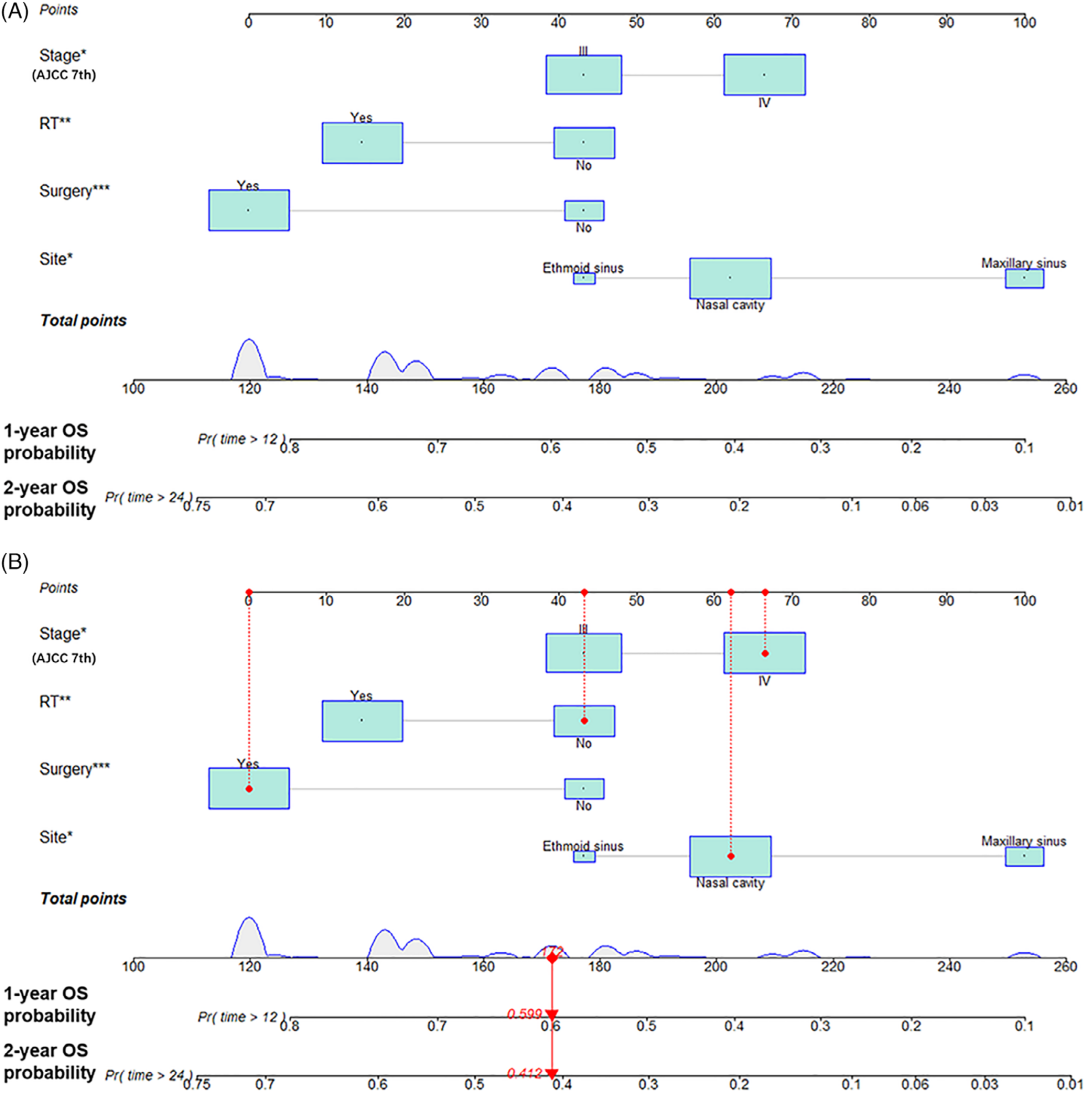
Nomogram predicting 1‐ and 2‐year OS probabilities in SMM patients. (A) Primary site, AJCC stage (seventh), RT, and surgery were identified as risk factors with the number of points indicated on the nomogram. Each category was assigned a certain number of points (top row) ranging from 0 to 100. The relevant scores for each category are presented in Table S2. For SMM patients suitable for using the nomogram, the points allocated to each variable are summed, and the total score is related to the individual's OS probabilities at 1 and 2 years. (B) Example of using the nomogram in a patient who was randomly selected from the training set. The patient had a primary tumor located in the nasal cavity (points = 62), AJCC clinical stage IV (points = 66), did not receive RT (points = 43), and received surgical treatment (points = 0). This patient's total score was 172, which corresponds to OS probabilities of 59.9% at 1 year and 41.2% at 2 years. AJCC, American Joint Committee on Cancer; OS, overall survival; RT, radiotherapy; SMM, sinonasal melanoma

Univariate Cox regression was performed to investigate potential risk factors for OS in SMM patients using the training cohort (Table 2). Variables with *p*‐values of <.05 in log‐rank tests were considered as candidate predictors for OS. Negative factors included T4 stage (HR 1.632, 95% CI 1.118–2.382, *p* = .011), positive cervical lymph node (HR 2.299, 95% CI 1.324–3.990, *p* = .003), distant metastasis (HR 2.334, 95% CI 1.381–3.943, *p* = .002), AJCC clinical stage IV (HR 1.995, 95% CI 1.353–2.943, *p* < .001), Ballantyne's stage II (HR 2.029, 95% CI 0.968–4.249, *p* = .061), and Ballantyne's stage III (HR 2.470, 95% CI 1.454–4.196, *p* = .001). In contrast, positive prognostic factors were primary disease in the nasal cavity (HR 0.410, 95% CI 0.255–0.660, *p* < .001), RT (HR 0.514, 95% CI 0.351–0.751, *p* = .001), and surgical treatment (HR 0.373, 95% CI 0.244–0.571, *p* < .001). These candidate variables were then included in the multivariable Cox regression analysis with a backward stepwise selection method to develop a model with the smallest AIC value to identify independent prognostic factors. Finally, a multivariate Cox regression model with the smallest AIC (AIC 972.3) was established to incorporate the best subsets of clinical variables, which comprised primary site in the ethmoid sinus (HR 0.349, 95% CI 0.141–0.866, *p* = .023) and nasal cavity (HR 0.495, 95% CI 0.299–0.819, *p* = .006), AJCC clinical stage IV (HR 1.539, 95% CI 1.003–2.363, *p* = .049), RT (HR 0.589, 95% CI 0.395–0.878, *p* = .009), and surgery (HR 0.449, 95% CI 0.284–0.711, *p* = .001) (Table 2). A nomogram integrating these variables was developed to predict the 1‐ and 2‐year OS probabilities in SMM patients (Figure 2A). An example illustrating the use of the nomogram is presented in Figure 2B. The relevant values for each variable in the nomogram are summarized in Table S2.

**TABLE 2:** Uni‐ and multivariate Cox analysis for prognostic factors on overall survival of SMM patients in the training cohort

Variables	Category	Univariate		Multivariate	
		HR (95% CI)	*p* value	HR (95% CI)	*p* value
Age	<50	reference	‐	‐	‐
	50–70	1.408(0.554–3.580)	.472	‐	‐
	>70	1.875(0.753–4.669)	.177	‐	‐
Sex	Female	reference	‐	‐	‐
	Male	0.981(0.674–1.429)	.922	‐	‐
Race	Asian/Pacific Islander	reference	‐	‐	‐
	Black	2.520(0.879–7.221)	.085	‐	‐
	White	1.297(0.601–2.799)	.507	‐	‐
Marital status	Single	reference	‐	‐	‐
	Married	0.868(0.411–1.832)	.709	‐	‐
	Others	1.614(0.762–3.418)	.211	‐	‐
Pathology	Melanoma (ICD‐O‐3 code 7020)	reference	‐	‐	‐
	Other pathology subtypes^a^	0.628(0.231–1.707)	.361	‐	‐
Multiple tumor history	No	reference	‐	‐	‐
	Yes	0.856(0.582–1.258)	.428	‐	‐
Site	Maxillary sinus	reference	‐	‐	‐
	Ethmoid sinus	0.507(0.255–0.660)	.126	0.349(0.141–0.866)	.023
	Nasal cavity	0.410(0.255–0.660)	<.001	0.495(0.299–0.819)	.006
T stage	T3	reference	‐		‐
	T4	1.632(1.118–2.382)	.011	‐	‐
N classification	N0	reference	‐	‐	‐
	N1	2.299(1.324–3.990)	.003	‐	‐
	Nx	2.381(0.583–9.724)	.227	‐	‐
M classification	M0	reference	‐		‐
	M1	2.334(1.381–3.943)	.002	‐	‐
AJCC clinical stage (seventh)	III	reference	‐	‐	‐
	IV	1.995(1.353–2.943)	<.001	1.539(1.003–2.363)	.049
Ballantyne's stage	I	reference	‐	‐	‐
	II	2.029(0.968–4.249)	.061	‐	‐
	III	2.470(1.454–4.196)	.001	‐	‐
RT	No	reference	‐	reference	‐
	Yes	0.514(0.351–0.751)	.001	0.589(0.395–0.878)	.009
Surgery	No	reference	‐	reference	‐
	Yes	0.373(0.244–0.571)	<.001	0.449(0.284–0.711)	.001
Chemotherapy	No	reference	‐	‐	‐
	Yes	0.838(0.437–1.607)	.595	‐	‐

*Note*: Two‐sided *p* < .05 was considered statistically significant.

Abbreviations: AJCC, American Joint Committee on Cancer; CI, confidence interval; HR, hazard ratio; OS, overall survival; RT, radiotherapy; SMM, sinonasal melanoma.

^a^ ICD‐O‐3 codes: 8722, 8770, 8771, 8772.

### 3.3 Nomogram validation and model performance

The performance of the nomogram was internally cross‐validated by estimating its discriminative and calibration abilities. The bias‐corrected C‐index (0.719, 95% CI 0.670–0.768) generated by bootstrapping with 1000 resamples was significantly superior to the Cox regression model developed using the seventh edition of the AJCC staging system (0.614, 95% CI 0.565–0.663, *p* < .001) and Ballantyne's stage classification (0.553, 95% CI 0.514–0.592, *p* < .001). Similar results were also observed in the validation cohort as the C‐index (0.682, 95% CI 0.605–0.759) was greater than that of the AJCC staging system (0.588, 95% CI 0.516–0.660, *p* = .015) and Ballantyne's stage classification (0.576, 95% CI 0.521–0.631, *p* = .007). These results indicate the new model shows better discriminative ability than conventional stage stratifications.

**FIGURE 3:**
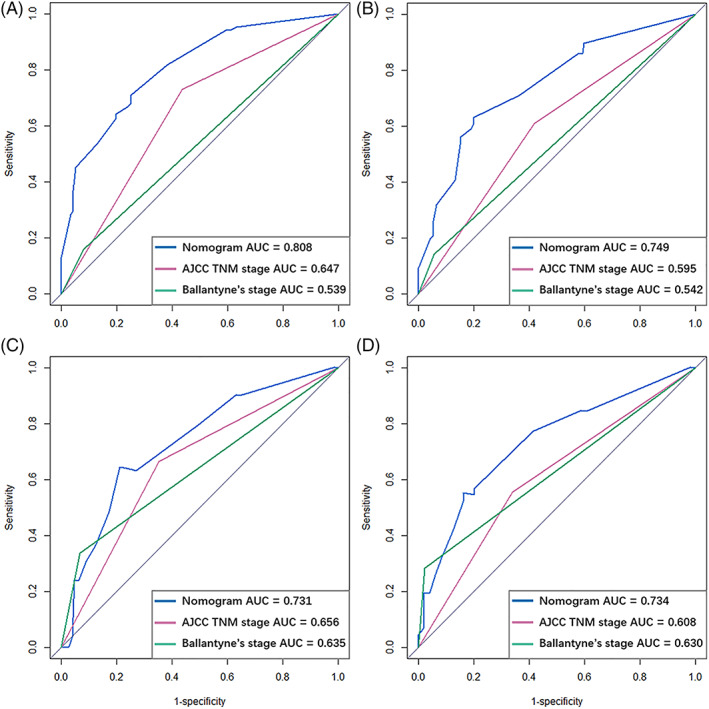
Time‐dependent ROC curves for the nomogram, AJCC staging system (seventh), and Ballantyne's stage to predict 1‐year (A) and 2‐year (B) OS in training set, and 1‐year (C) and 2‐year (D) OS in the validation set. (A) For predicting 1‐year OS in the training set, the AUC for the nomogram (0.808) is greater than the AUC for the Cox regression models established with the AJCC staging system (0.647) and Ballantyne's stage classification (0.539), indicating that the nomogram displays better accuracy for predicting 1‐year OS in SMM patients. (B) For predicting 2‐year OS in the training set, the AUC of the nomogram (0.749) is greater than that of the Cox regression models established using the AJCC staging system (0.595) and Ballantyne's stage classification (0.542). (C) For predicting 1‐year OS in the validation set, the AUC of the nomogram (0.731) is greater than that of the Cox regression models established using the AJCC staging system (0.656) and Ballantyne's stage classification (0.635). (D) For predicting 2‐year OS in the validation set, the AUC of the nomogram (0.734) is greater than that of the Cox regression models established with the seventh AJCC staging system (0.608) and Ballantyne's stage classification (0.630). These results demonstrate that the nomogram shows better performance for predicting the 1‐ and 2‐year OS of SMM patients. AJCC, American Joint Committee on Cancer; AUC, area under the curve; OS, overall survival; ROC, receiver operating characteristic; SMM, sinonasal melanoma

**FIGURE 4:**
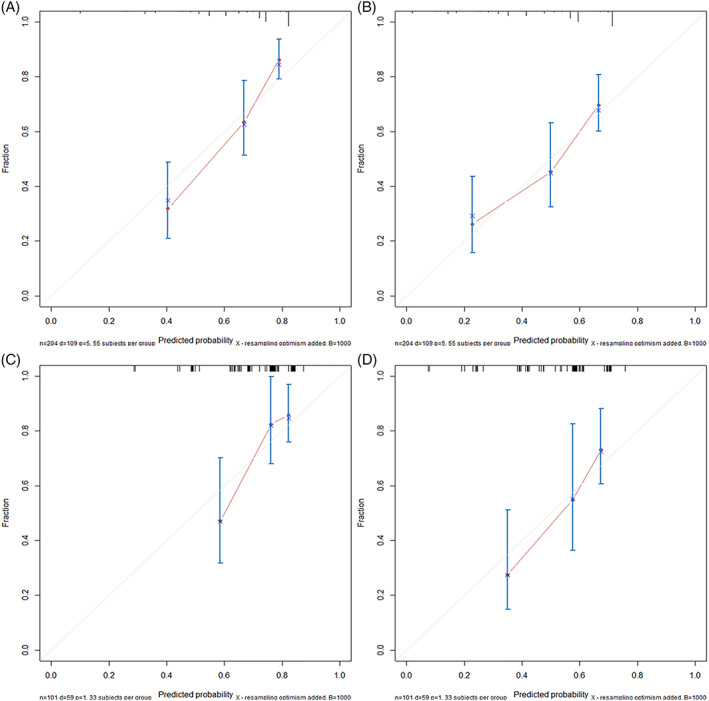
Calibration plots of the nomogram using cross‐validation with the bootstrap resampling method. (A,B) Calibration plots of the nomogram for predicting 1‐year OS (A) and 2‐year OS (B) using the bootstrap method with 1000 resamples in the training set. (C,D) Calibration plots of the nomogram for predicting 1‐year OS (C) and 2‐year OS (D) using the bootstrap method with 1000 resamples in the validation set. The calibration plots depict the calibration of the nomogram in terms of the agreement between the predicted OS risk (*x*‐axis) and the actual OS rate (*y*‐axis). The diagonal gray line represents perfect prediction with an ideal model. The pink solid line is the calibration curve representing the nomogram's performance. A closer fit to the diagonal gray line indicates better predictive ability. Overall, the calibration plots indicate that the nomogram displays acceptable predictive accuracy for 1‐ and 2‐year OS in SMM patients. OS, overall survival; SMM, sinonasal melanoma

**FIGURE 5:**
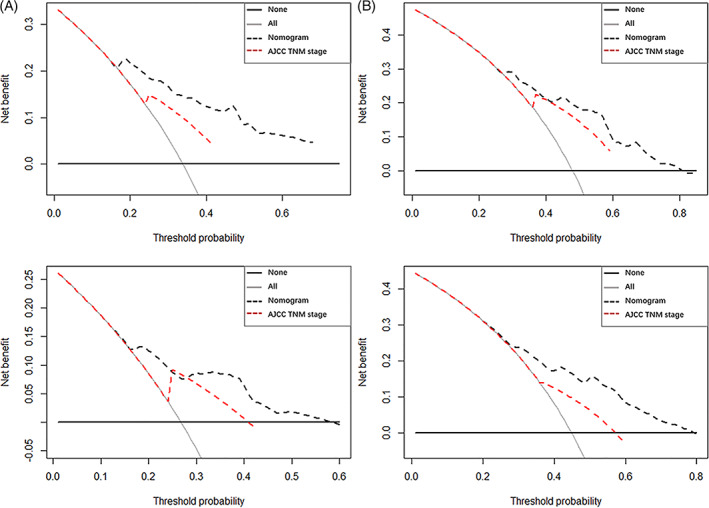
DCA of the nomogram relative to the Cox regression model established using the conventional AJCC staging system (seventh). DCA plots for the prediction performance of the nomogram compared with the conventional AJCC clinical stage (seventh) for 1‐year OS (A) and 2‐year OS (B) in the training set and for 1‐year OS (C) and 2‐year OS (D) in the validation set. Black horizontal line: assumes no patient will die. Gray line: assumes all patients will die. Dashed black line: prediction model of the nomogram. Dashed red line: prediction model established with the conventional AJCC staging system (seventh). Overall, the graphs show that the nomogram increases the expected net benefit of predicting survival outcomes compared with the conventional AJCC staging system at realistic threshold probabilities. AJCC, American Joint Committee on Cancer; DCA, decision curve analysis; OS, overall survival

Time‐dependent ROC curves were plotted, and the corresponding AUC values were calculated to compare the predictive abilities of the nomogram and the conventional stage classifications. The results indicate that the nomogram showed better predictive accuracy than the conventional stage systems with respect to the 1‐ and 2‐year OS rates (Figure 3). The ROC analysis of the new model demonstrated reliable predictive ability for the 1‐ and 2‐years OS rates, with AUC values reaching 0.808 (Figure 3A) and 0.749 (Figure 3B), respectively, in the training set. Similarly, the corresponding AUC values for predicting the 1‐ and 2‐year OS rates in the validation set were 0.731 (Figure 3C) and 0.734 (Figure 3D). The calibration plots validated by bootstrap resampling also demonstrated the reliability of the nomogram for predicting the 1‐ and 2‐year OS rates in both the training set (Figure 4A,B) and the validation set (Figure 4C,D). The results of DCA demonstrated that, across a wide range of threshold probabilities, using the nomogram to predict the 1‐ and 2‐year OS rates conferred greater benefit than the conventional staging systems in the training set (Figure 5A,B) and the validation set (Figure 5C,D). The nomogram also exhibited greater predictive performance than the AJCC staging system and Ballantyne's stage classification, with statistically significant, positive NRI and IDI values for predicting the 1‐ and 2‐year OS rates in the training set. Similar results were also observed in the validation set. The NRI and IDI values are presented in Table 3 and the improvement in integrated discrimination is presented as plots in Figure S2A,B for the training set and Figure S2C,D for the validation set.

**TABLE 3:** The results of IDI and NRI when comparing the nomogram with the AJCC staging system (seventh)

Category	NRI (95% CI)	*p* value	IDI (95% CI)
Training cohort			
1‐year OS	0.147(0.057–0.248)	.008	0.423(0.202–0.585)
2‐year OS	0.101(0.033–0.190)	.004	0.263(0.131–0.464)
Validation cohort			
1‐year OS	0.075(0.009–0.206)	.018	0.265(−0.001 to –0.465)
2‐year OS	0.113(0.0370–0.236)	.002	0.327(0.075–0.521)

*Note*: Two‐sided *p* < .05 was considered statistically significant.

Abbreviations: AJCC, American Joint Committee on Cancer; IDI, integrated discrimination improvement; NRI, net reclassification improvement; OS, overall survival.

## 4 DISCUSSION

SMM accounts for just 1% of all cases of melanoma and less than 1% of head and neck neoplasms.^1^, ^15^ Because of its rarity, a uniform staging system and standard treatment modalities have not yet been established for SMM.^10^, ^16^ Nomograms provide a pictorial representation of a regression formula, to integrate diverse prognostic and determinant variables.^17^ The points corresponding to each variable in the nomogram are simply summed, and the resulting total score is related to the patient's individual probability of a specified clinical event. With this easy‐to‐use pictorial scoring system, clinicians and patients can make an individualized prediction of the risk of a clinical outcome, fitting the concept of personalized medicine.^14^ In the current study, we investigated the risk factors that influence the prognosis of SMM and developed a prediction model, which was visualized using a nomogram, based on the population‐based SEER registry. Although prognostic nomograms for predicting OS and cancer‐specific survival (CSS) in patients with head and neck mucosal melanoma have been established in a recently published study,^18^ our novel nomogram took adjuvant treatment into account to evaluate prognostic outcomes in a more comprehensive approach.

The 1‐, 2‐, and 5‐year OS rates reported in our study were 66.2%, 52.0%, and 25.5%, respectively, which are consistent with the 5‐year OS rates of 20%–40% reported in previous studies.^8^ Cervical lymph node involvement and distant metastasis were identified as significant factors, although not independent negative prognostic markers, in the survival and univariate Cox analysis. These results are similar to those of another population‐based study of nodal and distant metastases in SMM performed by Low et al.,^19^ although the adverse impact of N+ neck status on prognosis was not confirmed in a systematic review conducted by Pontes et al.^20^ Moreover, in our study, patients with Ballantyne's stage II and III exhibited significantly decreased 1‐year (stage II: 47.7%; stage III: 50.0%) and 2‐year (stage II: 38.2%; stage III: 20.0%) OS rates compared with stage I, similar to the results reported by Chan et al.^21^ Whereas nodal and distant metastasis were commonly found during surveillance,^19^ cervical metastasis were rarely detected at initial diagnosis, with a reported frequency of about 10%.^3^, ^22^, ^23^ Therefore, the benefit of neck dissection or cervical irradiation as part of the primary treatment of SMM remains unclear.

In the current study, SMM was located in the nasal cavity in most patients in both cohorts, with fewer cases of SMM arising from the ethmoid sinus. Patients with SMM originating in the paranasal sinuses showed inferior survival compared with patients with tumors originating in the nasal cavity. Our results agree with another analysis of the SEER registry regarding the site of involvement^24^ as well as the results of earlier single‐center studies.^23^, ^25^, ^26^ Mucosal melanoma of the craniofacial region shows a tendency towards involvement of the skull base, orbit, or brain.^27^ Tumors with epicenters in the paranasal sinuses were more likely to show skull base or orbital invasion because of the anatomic proximity of the tumor to these critical structures.^28^ This poses a challenge to balance the need for radical resection with negative margins and protecting vital structures to preserve their functions.

Surgical treatment is the mainstay approach for patients with mucosal melanoma.^29^ Patients who did not undergo surgical treatment showed significantly worse survival in the current study, with a 1‐year OS rate of just 26.7% compared with 76.4% in patients who underwent surgery. Extensive excision of the primary disease with negative margins has been proposed to improve the prognosis of mucosal melanoma.^27^ Intranasal endoscopic resection, open maxillectomy, and craniofacial resection are the most common surgical approaches for patients with SMM.^30^ Sayed et al.^30^ reported that patients who underwent craniofacial resection had worse OS than patients who underwent other surgical approaches, whereas the outcomes were similar between patients who underwent open maxillectomy or endoscopic resection, consistent with the study by Lundberg et al.^31^ The role of endoscopic resection has yet to be widely acknowledged because of the difficulty of achieving complete resection with clear margins, despite its advantages, including minimal complications and better protection of vulnerable structures. Gillian et al^7^ proposed that endoscopic surgery should be reserved for experienced surgeons.

Because of the high local aggressiveness of SMM and the complex anatomy of the sinonasal region, postoperative RT is commonly used as an adjuvant treatment to improve prognosis,^4^, ^26^, ^32^ especially in patients with advanced disease, wide extension, or positive margins.^16^ In the current study, surgery and RT were both independent prognostic factors and were integrated into the prediction nomogram. A meta‐analysis by Hu et al^11^ revealed that postoperative RT increased the 3‐year OS rate, although the improvements in local control and disease‐free survival were not statistically significant, consistent with the results of other studies.^16^, ^27^ However, Ajmani et al.^32^ argued that postoperative RT had a limited benefit on survival, regardless of margin status. Chemotherapy is generally administered to patients with SMM and distant metastasis or as palliative treatment for very advanced disease with unclear effectiveness.^4^, ^7^ The traditional standard chemotherapy regimen for metastatic melanoma used to be dacarbazine.^33^ Hahn et al.^29^ reported that chemotherapy contributed to better outcomes in patients with mucosal melanoma, although chemotherapy did not improve prognosis in our study, even in patients with advanced‐stage SMM. Despite the ill‐defined overall survival benefit of chemotherapy in SMM patients, it still offers palliative treatment options for advanced disease, and represents a common salvage regimen for refractory melanoma and tumors harboring no somatic mutations that in lack of specific inhibitors, confirming its role as applicable second‐line adjuvant treatment for SMM.^34^

Although the medical records provided in SEER database did not include information about implementation of immunotherapy, our nomogram might help identify patients with extremely poor prognosis, such as patients whose expected 2‐year survival possibility were less than 20% for example, who usually suffered from untreated metastatic or inoperable disease and were indicated for immunotherapies. The combination of nivolumab (anti‐PD‐1) and ipilimumab (anti‐CTLA‐4) is amendable to patients with advanced melanoma as the current standard of care, which might improve the 5‐year survival rate to approximately 50%.^35^ However, the clinical benefit of various kinds of targeted agents and immunotherapies for treating mucosal melanoma has not been thoroughly confirmed, indicating that more randomized controlled trials regarding treatment outcomes of immunotherapies were in need for exclusively SMM patients.^36^ The nomogram constructed in our study might serve as supplementary means to identify potential candidates for immunotherapy trials, which might be of unique clinical value for rare diseases such as SMM.

The current TNM staging system for mucosal melanoma of the head and neck was proposed in 2009 in the seventh edition of the AJCC Cancer Staging Manual. Advanced stage was significantly associated with poor outcomes in our study, consistent with prior studies.^3^, ^10^, ^23^, ^28^ However, the predictive accuracy of the AJCC staging system for survival is restricted because the classification covers only a few aspects of anatomical tumor extension. In this study, by incorporating the AJCC staging system with other significant clinical predictors associated with prognosis, including primary site and treatment modalities, our nomogram was based on a scoring system that was more accurate and individualized in predicting patient survival outcomes than conventional, descriptive anatomical staging systems.

Several limitations should be acknowledged. First, this study was limited by its retrospective nature. Second, we could not collect data about RT dose, chemotherapy regimens, surgical approach, and margin status from the SEER registry. Variations in these factors could influence the significance of the present results and affect the validity of the nomogram. In particular, information on immunotherapy and targeted therapy was unavailable, which might reduce the predictive validity of the nomogram for advanced‐stage SMM, because the model used to develop the nomogram did not include variables for systemic immunotherapy. Biological and immunomodulatory oncological interventions have joined widely accepted interventions for the management of cutaneous melanoma, and the expanding role of immunotherapy in mucosal melanoma might alter the treatment paradigm for SMM and thus limit the utility of our nomogram in the near future. Third, the stage classification and WHO classification of pathological subtypes of mucosal melanoma in the head and neck have been updated several times in recent years, and these changes might influence our results. Moreover, comparing staging systems with this nomogram has some limitations. Integrating the AJCC staging system with the treatment options in this nomogram may decrease its utility for initial evaluation of patients at presentation because staging systems can be used to guide treatment decision‐making, not just to determine prognosis. Moreover, our results showed that patients who did not undergo surgical treatment had significantly worse OS. However, the absence of surgical treatment was very likely to be positively correlated with advanced stage of disease, which could deteriorate the prognostic outcomes of these SMM patients, causing selection bias that might attenuate the reliability of this prognostic model. Furthermore, the nomogram was constructed only on OS, and no other clinical outcomes. This was mainly because when we tried to build Cox regression models based on other outcomes including disease‐free survival (DSS), some variables in the regression models were always found with lack of statistical significance, probably given the small sample size. For instance, we found two variables including clinical stage and RT were not statistically significant for DSS in the Cox regression model, precluding the creation of a nomogram on this outcome. Finally, although about 28% of the US populations were covered by the SEER 18 registry, the cohort used in our study was still relatively small, which may be because of the rarity of SMM. Although the nomogram was internally validated with cross‐validation and bootstrapping methods, it was established using the SEER registry and was not validated externally with patients from other centers or another database, which could alleviate data overinterpretation but would not completely avoid it due to possible overfitting. Thus, we cannot assess the transferability or generalizability of our results to different patient populations. Further studies are necessary to investigate the external validity and universal applicability of this nomogram.

## 5 CONCLUSION

In this study, we developed a nomogram to predict the probability of 1‐ and 2‐year survival rates in patients with SMM using data from the population‐based SEER registry. The nomogram integrated the AJCC staging system with tumor site, surgery, and RT, and showed reliable discriminative ability and predictive accuracy relative to conventional clinical stage classifications. The nomogram could be used to predict the survival probability at different time points for individual patients with SMM.

## CONFLICT OF INTEREST

The authors have no funding, financial relationships, or conflicts of interest to disclose.

## Supporting information


**FIGURE S1** Kaplan–Meier plots of OS in SMM patients in the training set stratified by AJCC tumor stage (seventh) (A), AJCC node classification (B), AJCC metastasis classification (C), and chemotherapy (D). AJCC, American Joint Committee on Cancer; OS, overall survival; SMM, sinonasal melanoma

**FIGURE S2** Graphical depiction of the IDI to reflect the overall improvement in the predictive accuracy using the nomogram instead of the conventional AJCC staging system (seventh) for predicting 1‐year OS (A) and 2‐year OS (B) in the training set, and 1‐year OS (C) and 2‐year OS (D) in the validation set. The red color blocks represent the extent of improvement in the predictive accuracy using the nomogram, whereby a greater area indicates greater improvement of the nomogram compared with the AJCC clinical stage. AJCC, American Joint Committee on Cancer; IDI, integrated discrimination improvement; OS, overall survival

**TABLE S1** Comparison of 1‐ and 2‐year OS rates in SMM patients stratified by clinical characteristics in the training set. OS, overall survival; SMM, sinonasal melanoma

**TABLE S2** Scoring system for each variable included in the nomogram
