# Supplementary figures and images for: A nomogram for predicting overall survival of patients with sinonasal melanoma: A population‐based study
Source: Laryngoscope Investig Otolaryngol. 2022 Nov 22;7(6):1837–48. doi: 10.1002/lio2.951 (PMC9764764; doi:10.1002/lio2.951)

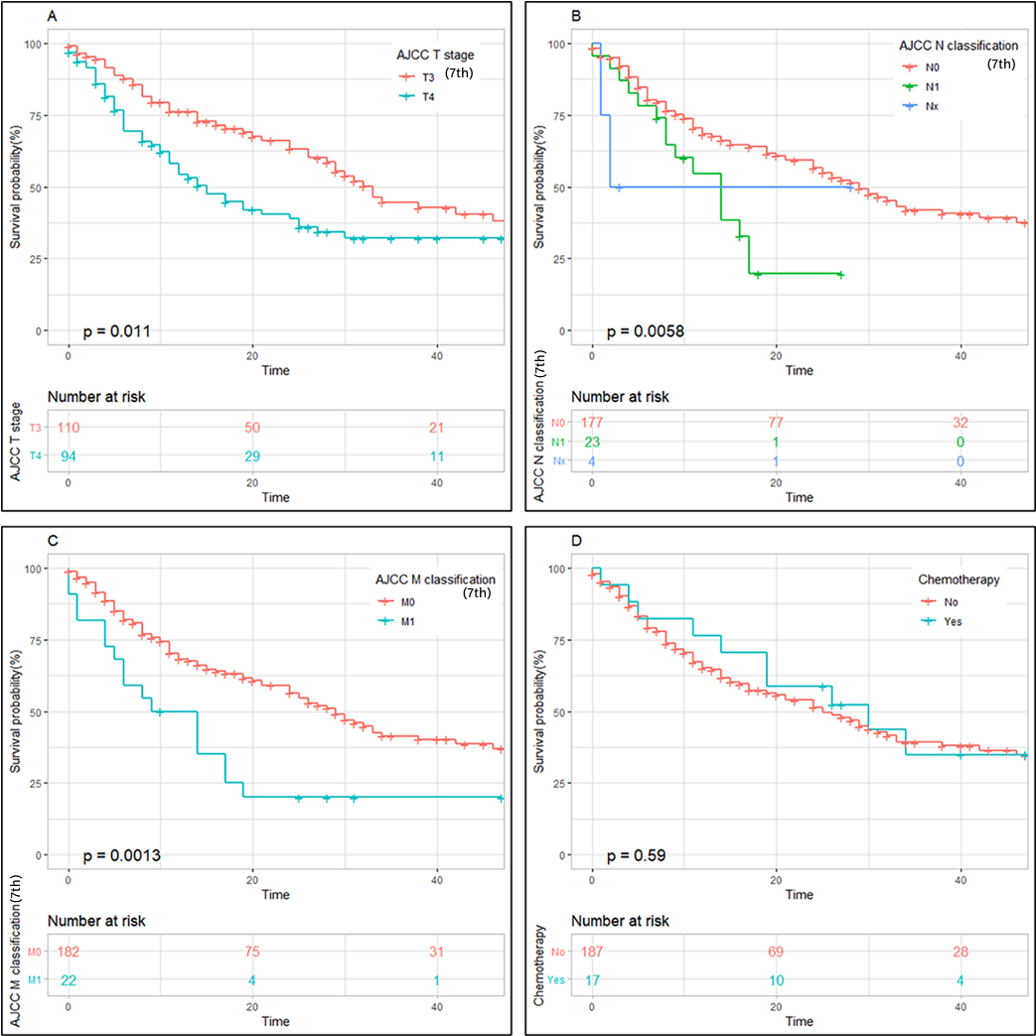

Supplement: Supplementary file 1 — FIGURE S1 Kaplan–Meier plots of OS in SMM patients in the training set stratified by AJCC tumor stage (seventh) (A), AJCC node classification (B), AJCC metastasis classification (C), and chemotherapy (D). AJCC, American Joint Committee on Cancer; OS, overall survival; SMM, sinonasal melanoma [file LIO2-7-1837-s003.tif]

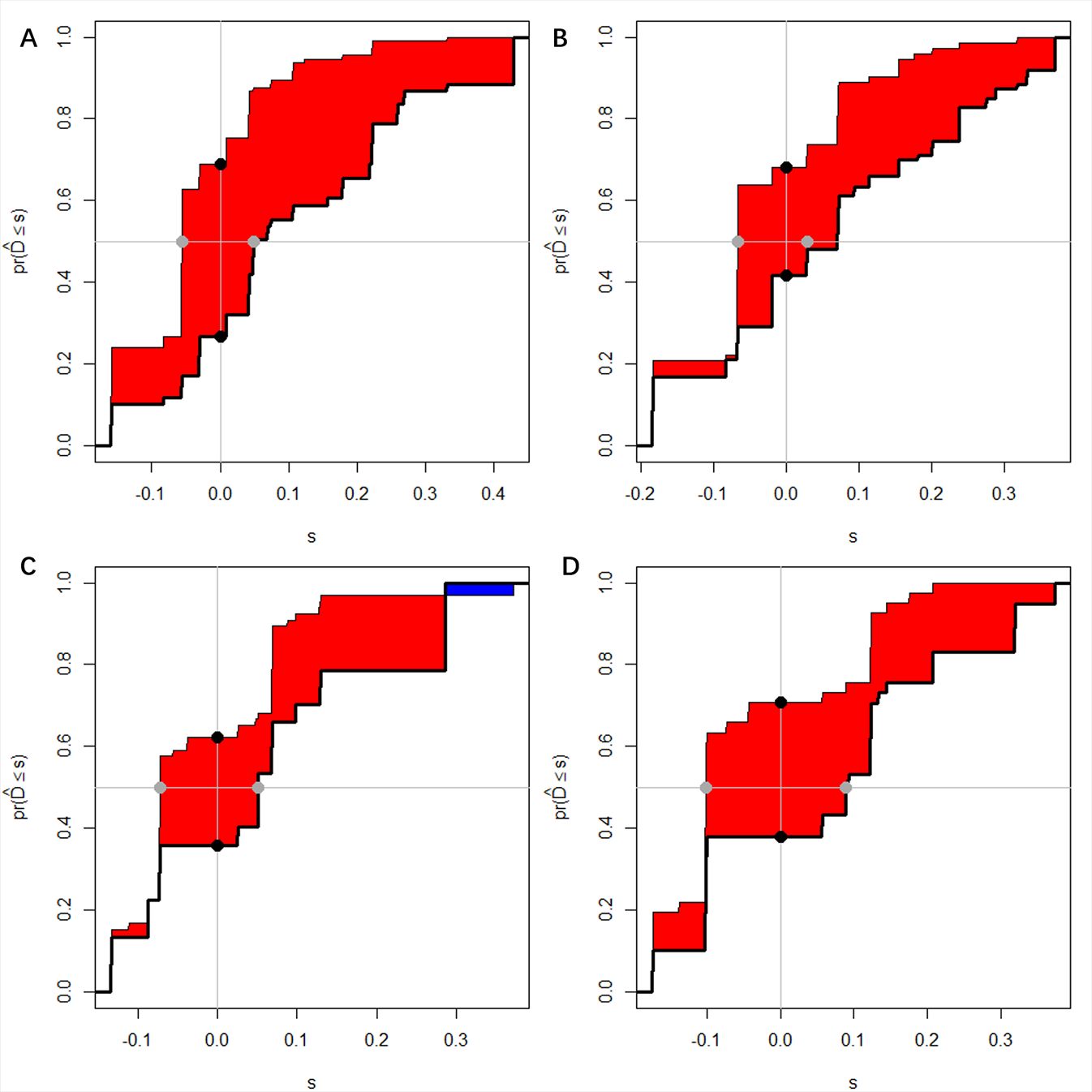

Supplement: Supplementary file 2 — FIGURE S2 Graphical depiction of the IDI to reflect the overall improvement in the predictive accuracy using the nomogram instead of the conventional AJCC staging system (seventh) for predicting 1‐year OS (A) and 2‐year OS (B) in the training set, and 1‐year OS (C) and 2‐year OS (D) in the validation set. The red color blocks represent the extent of improvement in the predictive accuracy using the nomogram, whereby a greater area indicates greater improvement of the nomogram compared with the AJCC clinical stage. AJCC, American Joint Committee on Cancer; IDI, integrated discrimination improvement; OS, overall survival [file LIO2-7-1837-s002.tif]
